# Structural and physicochemical characteristics of wheat starch as influenced by freeze-thawed cycles and antifreeze protein from *Sabina chinensis* (Linn.) Ant. cv. Kaizuca leaves

**DOI:** 10.1016/j.fochx.2023.100927

**Published:** 2023-10-05

**Authors:** Mei Liu, Jie Li, Hao Ma, Guolan Qin, Mengge Niu, Xiaoyin Zhang, Jin Zhang, Yangkun Wei, Jiajing Han, Ying Liang, Shenying Zhang, Lulu Yin, Haojia Zhu, Ying Huang, Limin Li, Xueling Zheng, Chong Liu

**Affiliations:** a National Engineering Laboratory/Key Laboratory of Henan Province, Henan University of Technology, Zhengzhou 450001, China; b College of Food Science and Engineering, Henan University of Technology, Zhengzhou 450001, China; c College of Biological Engineering, Henan University of Technology, Zhengzhou 450001, China

**Keywords:** Freeze-thawed cycles, Antifreeze protein, *Sabina chinensis* (Linn.) Ant. cv. Kaizuca leaves, Wheat starch, Physicochemical characteristics, Semi-crystalline granular structures

## Abstract

The effects of freeze-thawed cycles (FTs) and a new antifreeze protein from *Sabina chinensis* (Linn.) Ant. cv. Kaizuca leaves (ScAFP) on the structure and physicochemical characteristics of wheat starch were studied. The mechanical breaking exerted by ice crystals on starch granules during FTs gradually deepened, sequentially squeezing the surface (2–6 FTs), amorphous region (8 FTs) and crystalline region (10 FTs) of starch granules. These changes led to reduced thermal stability, increased retrogradation tendency, and weakened gel network structure. The addition of ScAFP retarded the damage of ice crystals on starch granule structure and crystal structure during FTs, and significantly reduced the retrogradation tendency. Compared with native starch, the hardness of freeze-thawed starch without and with added ScAFP after 10 FTs decreased by 17.85% and 9.22%, respectively, indicating ScAFP improved the gel texture properties of freeze-thawed starch. This study provides new strategies for improving the quality of frozen starch-based foods.

## Highlights

• Mechanical damage to starch granules deepened as freeze–thaw cycles increased.
• ScAFP retarded the damage of ice crystals on granular and crystal structure of starch.
• ScAFP improved thermal stability and gel texture properties of freeze-thawed starch.

## 1 Introduction

As a key technology of quick-frozen flour products, frozen dough has promising prospects due to its convenience, quickness, health and nutrition (Liu et al., 2018). However, the ice crystals recrystallization caused by temperature variation during long-term storage and transportation aroused the deterioration in the physicochemical properties of main components (gluten, yeast and starch) in frozen dough (Meziani et al., 2012, Tao et al., 2015, Wang et al., 2015), eventually leading to the exacerbation of dough properties and its product quality. Because of this, the large-scale application of frozen dough was limited (Luo, Sun, Zhu, & Wang, 2018).

Starch, as the main component of dough, has a nonnegligible impact on the quality of final flour products (Huang, Wang, Fan, & Ma, 2022). Wheat starch is mainly composed of highly organized semi-crystalline granular structures, including crystalline and amorphous regions. The crystalline region is primarily formed by amylopectin molecules in a double helix structure, which is dense and less susceptible to external forces, while the amorphous region is mainly formed by amylose molecules in a loose structure, which is susceptible to external forces (Huang et al., 2022, Villwock and BeMiller, 2022). Due to the inconsistencies in composition and structure, there may be differences in the tolerance of the crystalline and amorphous regions to ice crystal formation and recrystallization. It has been studied that freezing and freeze-thawed cycles (FTs) led to an increase in the damaged starch and leachate contents of wheat starch, altered the granule morphology, and significantly raised the pasting viscosity of starch (Liu et al., 2022, Tao et al., 2015). Starch isolated from dough with multiple freeze-thawed cycles showed holes and cracks on the surface of starch granules, and multi-scale structure of starch was destroyed (Wang et al., 2022). Current researches have shown that frozen storage treatments significantly alter the structure and physicochemical properties of starch, but the mechanisms of starch properties deterioration during frozen storage are not well understood. Thereby, it is important to investigate the action mechanism of ice crystals on starch granules during frozen storage and weaken the damage of ice crystals on starch granules for the quality improvement of frozen starch-based foods.

Antifreeze proteins (AFPs), also referred as ice-structuring proteins or thermal hysteresis proteins, protect food from freezing by lowering the freezing point of low-temperature organisms in a non-equilibrium pattern. The affinity of antifreeze protein for the surface of ice crystals enables it to bond with ice crystals during temperature fluctuations, inhibit recrystallization of ice crystals, modify ice crystal morphology, and improve ice crystal stability (Ding et al., 2020, Liu et al., 2018). These distinctive characteristics make AFPs an excellent candidate for frozen food protectants. Especially the plant AFPs, exhibiting excellent inhibiting ability of ice crystals recrystallization, can enhance the quality of frozen foods in long-term frozen storage (Chen et al., 2021). There have been many reports on the improvement of frozen dough and its components (gluten) by plant AFPs from different sources, such as oat (Zhang, Zhang, Wang, Qian, & Qi, 2015), barley (Ding et al., 2020), carrot (Zhang, Zhang, & Wang, 2007) etc., however, the influence of plant AFPs on the structural and physicochemical characteristics of frozen wheat starch have not been evaluated yet. Considering the indispensable role of starch playing in guaranteeing the quality of flour product, it is necessary to explore the effects of plant AFPs on the structural and physicochemical characteristics of starch during frozen storage. In our previous work, a new plant AFP, with a molecular weight of 35.7 kDa and a thermal hysteresis activity of about 0.44 °C, was extracted from *Sabina chinensis* (Linn.) Ant. cv. Kaizuca leaves (ScAFP) (unpublished data). ScAFP has potential applications as an ice crystal growth inhibitor to protect the quality of frozen foods, but no relevant studies have been reported.

In this study, the granular morphology, particle size distribution, relative crystallinity, solubility, swelling power, pasting properties, thermal properties and gel texture properties of wheat starch were comprehensively investigated during freeze-thawed cycles. Meanwhile, a new plant AFP extracted from *Sabina chinensis* (Linn.) Ant. cv. Kaizuca leaves (ScAFP) was selected to systematacially investigate its effects on the structural and physicochemical characteristics of wheat starch during FTs. This study could enrich the quality deterioration mechanism of starch during FTs and further clarify the causes of quality deterioration of frozen wheat flour products from the perspective of starch; additionally, provide the theory basis for the application of plant AFPs in the quality improvement of frozen starch-based product in terms of structural and physicochemical characteristics of starch.

## 2 Materials and methods

### 2.1 Materials

Wheat starch (purity > 99 %) was purchased from Shanghai Lvyuan Starch Co., Ltd. ScAFP preparation: After cold-induced (−20 °C, one month) *Sabina chinensis* (Linn.) Ant. cv. Kaizuca leaves were pulverized under liquid nitrogen, mixed with Tris-HCl (62.5 mM, pH 6.8) in a 1:2 ratio and stirred for 1 h for extraction, and then centrifuged at 12,500×g, 4 °C for 30 min. The upper liquid layer was taken and mixed with pre-cooled acetone at a ratio of 1:4, and then precipitated at −20 °C for 12 h. The precipitate was centrifuged at 12,500×g, 4 °C for 20 min and lyophilized to obtain the crude ScAFP. The obtained crude ScAFP was redissolved in Tris-HCl and heated at 75 °C for 20 min, and the pure ScAFP with certain thermal stability could be obtained by electrophoretic protein band recovery.

### 2.2 Preparation of multiple FT-treated starch

The wheat starch was mixed with distilled water at a ratio of 1:1.5. ScAFP group was supplemented with 0.5 % ScAFP (optimal concentration selected based on previous experiments). The prepared starch suspensions was stirred for 30 min, transferred to −40 °C freezer for freezing treatment (2 h), and then, stored in −18 °C freezer for 22 h and thawed at 25 °C for 2 h. The frozen storage and thawing treatment were taken as a FT. After 2, 4, 6, 8 and 10 FTs, the sample with ScAFP added was centrifuged for 10 min at 4000×g, the precipitate was mixed with distilled water and carefully centrifuged at 4000×g for 10 min for twice to remove ScAFP. Following this, the precipitate was lyophilized and ground for further analysis. The samples without added ScAFP were subjected to the same FTs and centrifugation treatment.

### 2.3 Microstructure observation

Scanning electron microscopy (SEM) (S-3700 N, HITACHI, Tokyo, Japan) was used to observe the microscopic structure of starch. Samples were stuck on aluminum specimen stubs, and then coated with gold. All the samples were observed at 5 kV with ×1000 magnification (Liu et al., 2022).

### 2.4 Particle size distribution

The particle size and specific surface area of starch was determined by a laser particle size analyzer (BT-9300HT, Dandong, China) (Wang, Bai, Jiang, Lang, & Yu, 2019).

### 2.5 X-ray diffraction (XRD)

X-ray diffractograms of wheat starch were obtained with an X-ray diffractometer (D8 Advance, Bruker AXS, Germany) (Liu et al., 2022). The test parameters were as follows: radiation source Cu-Kα, voltage 40 kV, current 35 mA, scanning range 4°-40°, scanning speed 4°/min. The relative crystallinity (%) was determined as the ratio of diffraction peak area to the total diffraction area.

### 2.6 Solubility and swelling power

Solubility and swelling power were determined according to the method of Shang, Li, Zhao, Liu, and Zheng (2020). Weighed samples (W_0_, dry basis) were suspended in deionized water to prepare 2 % slurry (w/w). The slurry was placed in an oscillating water-bath (85 °C) for 30 min, and cooled to room temperature. Then, the slurry was centrifuged at 3000 r/min for 20 min. The supernatant was poured and dried to constant weight (W_1_) in an oven at 105 °C. The weight of residue was also recorded (W_2_). Solubility and swelling power were calculated based on Eqs. (1) and (2), respectively.

(1) Solubility (%) = W_1_/W_0_ × 100

(2) Swelling power (%) = W_2_/(W_0_ × (100 − Solubility)) × 100

### 2.7 Pasting properties

The pasting properties of starch were determined by using a Rapid Viscosity Analyzer (RVA; Newport Scientific, Pty Ltd., Australia）based on the method reported by Liu et al. (2022). The “Standard method 1” of the RVA heating program was selected, and 3 g of starch (with and without ScAFP treatment) subjected to different freeze-thawed cycles were weighed into 25 g of distilled water on a 14 % wet basis and stirred well to start the measurement.

### 2.8 Thermal properties

DSC-7 analyzer (Perkin Elmer, Norwalk, CT, USA) was used to determine the thermal properties of starch samples (Liu et al., 2022). Approximately 2.5 mg of starch sample was weighed in a crucible and 7.5 mg distilled water was added. The samples were sealed and allowed to equilibrate at room temperature for 24 h. A blank crucible was used as a reference, and then the crucible was heated from 20 °C to 90 °C at a rate of 10 °C/min. The onset temperature (T_o_), the endothermic peak temperature (T_p_), the conclusion temperature (T_c_) and the enthalpy of gelatinization (ΔH, J/g) were obtained. Three replications were measured for each experiment.

### 2.9 Gel texture properties

Gel texture properties were measured by TA-XT Plus texture analyzer (Stable Microsystems, Godalming, UK) (Guo, Wang, Liu, & Wang, 2020). The corresponding measurement parameters were as follows: probe P 0.5, pre-test, test and post-test velocities of 2.0 mm/s, 1.0 mm/s and 1.0 mm/s, respectively, trigger force 5.0 g, and 50 % strain.

### 2.10 Data analysis

All data are presented as the mean ± standard deviation of three replicates and were analyzed for one-way significance using SPSS software.

## 3 Results and discussion

### 3.1 Microstructure observation

**Fig 1:**
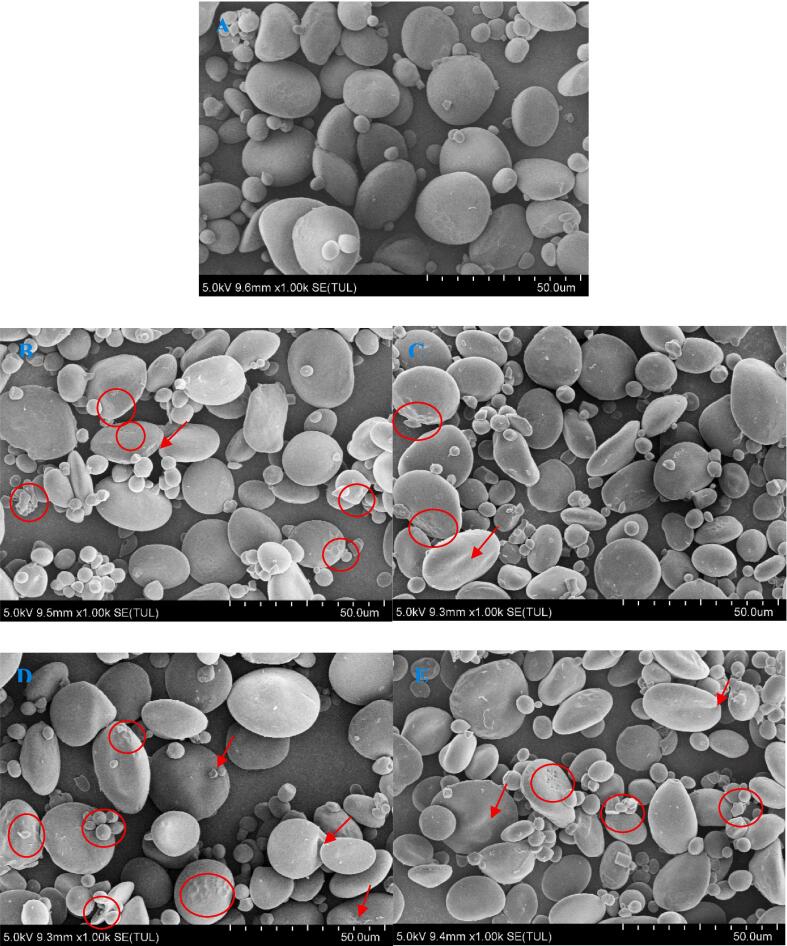
SEM photographs of starch with or without ScAFP added during multiple FTs. A, B, C, D, and E were native wheat starch, wheat starch without ScAFP after 6 FTs, wheat starch with ScAFP after 6 FTs, wheat starch without ScAFP after 10 FTs, and wheat starch with ScAFP after 10 FTs, respectively.

During FTs, ice crystals’ formation and recrystallization were repeatedly occurred inside and outside wheat starch granules. The wheat starch granules undergo a series of complex physical effects, and the most visualized physical effect suffered was the changes of its granular structure. Therefore, the granular structure of starch with and without ScAFP during FTs was investigated, as shown in Fig. 1. It can be seen from Fig. 1 that some pores, depressions and cracks emerged on the surface of freeze-thawed starch granules, which is in line with previous studies (Tao et al., 2015, Wang et al., 2022). It indicated that FTs resulted in mechanical damage to the surface or inside of starch granules. Some micropores on the surface of starch granules and a cavity structure inside it were observed, which could be explained by the expansion and compression exerted by the ice-matrix on the granules, occupying channels inside the granule or on its walls (Wang et al., 2022). Repeated FTs led to the generation of ice crystals expansion pressure in the internal and external starch particles, during which the water distribution, ice nucleation, growth and recrystallization induced the structural damage of starch particles.

Fewer micropores and depressions were observed on the surface of starch granules with ScAFP added, indicating that ScAFP could effectively mitigate the mechanical damage of starch granules during FTs. This may be attributed to the inhibition of ice crystal recrystallization during FTs with the addition of ScAFP, which reduced the compression of starch granules by ice crystals (Ding et al., 2020). The alternative explanation is that ScAFP may be able to adhere on the surface of starch granules to form a protective layer thus reducing the damage to starch granules by ice crystals (Gong et al., 2019).

### 3.2 Particle size distribution

The particle size distribution of wheat starch could reflect morphology of wheat starch granules and influences the baking properties of frozen dough (particularly A-type starch granules) (Tao, Wang, Wu, Jin, & Xu, 2016). The D10, D50 and specific surface area of starch with and without ScAFP during FTs were summarized in Table 1.

**Table 1:** D10, D50 and specific surface area of starch upon different FTs with and without ScAFP.

Samples	D10	D50	Specific surface area
NWS	4.716 ± 0.060^bc^	17.80 ± 0.03^d^	250.1 ± 1.7^ef^
CS-2c	4.579 ± 0.116^b^	17.64 ± 0.01^c^	253.2 ± 3.6^f^
CS-4c	4.034 ± 0.174^a^	17.15 ± 0.06^b^	271.8 ± 3.5^g^
CS-6c	3.964 ± 0.077^a^	16.97 ± 0.08^a^	279.0 ± 5.2^h^
CS-8c	4.624 ± 0.165^bc^	17.64 ± 0.09^c^	254.6 ± 4.6^f^
CS-10c	4.773 ± 0.126^c^	17.71 ± 0.10^cd^	248.4 ± 5.9^ef^
SS-2c	5.193 ± 0.045^d^	18.04 ± 0.05^e^	244.1 ± 1.1^de^
SS-4c	5.753 ± 0.033^f^	18.41 ± 0.03^g^	235.7 ± 2.1b^c^
SS-6c	6.165 ± 0.038^g^	18.66 ± 0.00^h^	227.8 ± 0.7^a^
SS-8c	5.798 ± 0.130^f^	18.45 ± 0.03^g^	229.8 ± 4.3^ab^
SS-10c	5.537 ± 0.060^e^	18.27 ± 0.09^f^	239.2 ± 2.2^cd^

Mean of three measurements ± standard deviation. Different lowercase letters in the same column indicate significant differences (*P* < 0.05) between different treatments. Abbreviations: D10, maximum diameter represented by 10 % of the granule size distribution; D50, maximum diameter represented by 50 % of the granule size distribution; specific surface area refers to the total area of the unit mass of starch granule. FTs: freeze-thawed cycles; NWS: native wheat starch; CS and SS represent the starch without and with ScAFP added, respectively; c in the first column represents freeze–thaw cycles, and different numbers represent the corresponding number of freeze–thaw cycles.

After 4 and 6 FTs, the D10 and D50 values of starch-control granules decreased significantly and the specific surface area raised significantly compared to native starch, indicating that the size of starch granules decreased after freeze-thawed treatment. This could be due to external ice crystal growth squeezing the starch granules, leading to damage and depression of the starch granules, or internal ice crystal growth creating a cavity structure resulting in greater shrinkage of the starch during the drying process (Su et al., 2020). However, compared to 6 FTs, the D10 and D50 values of starch increased and the specific surface area decreased after 8 and 10 FTs. This is probably because the aggregation of damaged starch granules after 8 and 10 FTs, resulting in an increase in starch granule size. This is similar to the results of previous study where aggregation of damaged potato starch granules occurred after freezing treatment (Szymońska, Krok, & Tomasik, 2000).

The D10 and D50 values of freeze-thawed starch with ScAFP added were higher significantly and the specific surface area was significantly lower compared to the control. It indicated that the addition of ScAFP had a significant protective effect on the structure of starch granules, leading to a significant increase in the size of freeze-thawed starch granules, which was consistent with the results of 3.1 and could be observed in the microstructural images.

### 3.3 X-ray diffraction (XRD)

**Fig 2:**
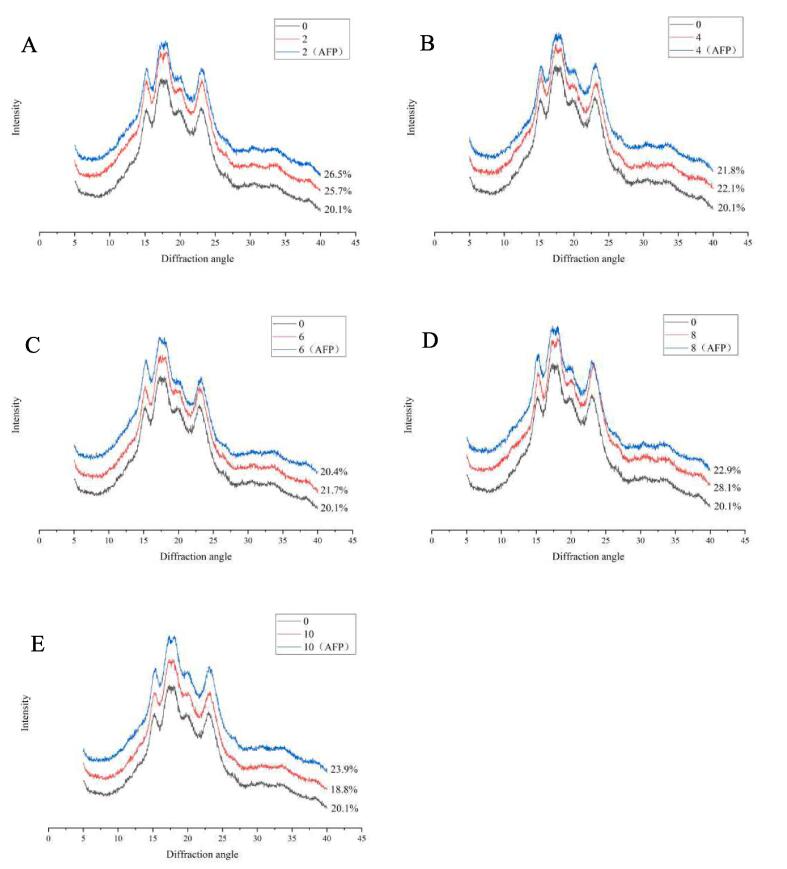
XRD patterns of native wheat starch and starch-ScAFP during multiple FTs. A, B, C, D, and E were XRD patterns of native wheat starch and starch-ScAFP after 2, 4, 6, 8, 10 FTs, respectively. Abbreviations: 0: native wheat starch; 2, 4, 6, 8, 10: different FTs-treated wheat starch; 2(AFP), 4(AFP), 6(AFP), 8(AFP), 10(AFP): different FTs-treated wheat starch with ScAFP added.

The X-ray diffraction patterns and relative crystallinity of wheat starch during FTs were presented in Fig. 2. All starch samples with or without added ScAFP exhibited strong diffraction peaks at ca. 15, 17, 18, and 23 (2θ), which is a typical A-type X-ray diffraction pattern (Shevkani, Singh, Bajaj, & Kaur, 2017), indicating that neither FTs nor the addition of ScAFP changed the crystalline type of starch.

The relative crystallinity of starch with or without ScAFP after 2 FTs was significantly higher than that of native starch. This indicated an increase in cavities and channels on the starch surface after 2 FTs (Section 3.1), which promotes water infiltration and leaching of free material (amylose, proteins and lipids) from the amorphous regions, leading to increased mobility and more rearrangement of amylopectin. It has been reported that amylose molecules may hinder the mobility of amylopectin molecules, which in turn limits the structural reorganization of starch (Cheetham & Tao, 1998). The relative crystallinity of starch without ScAFP gradually decreased with increasing FTs (2, 4, and 6 FTs). This indicated that water infiltration promoted ice crystals growth and recrystallization inside starch granules during FTs, which led to the breakage of hydrogen bonds inside starch, disruption of the double helix and microcrystalline structure. As a result, the relative crystallinity of starch was reduced. Compared to control, the crystallinity of starch-ScAFP did not obviously change.

After 8 FTs, due to the enhanced effect of ice crystals recrystallization, on the one hand, it may lead to the dehydration of starch granules, which reduces the damage of internal ice crystals to the ordered structure of starch. On the other hand, the larger squeezing pressure of the large ice crystals causes the amorphous region of the starch, which acts as a “shock absorber” (Perry & Donald, 2000), to be compressed and become compacted. This protected the crystalline structure of starch from the ice crystal compression and allowed the starch chains to stack more tightly, which promoted the rearrangement of amylopectin and led to a significant increase in the relative crystallinity of starch. The relative crystallinity of starch-ScAFP was significantly lower than that of control. This indicated that ScAFP bound with the external ice crystals of starch and decreased the compression damage of ice crystals to the amorphous region of starch, reduced the leaching of substances in the amorphous region, and inhibited the increase in the proportion of crystalline regions.

After 10 FTs, the relative crystallinity of starch decreased significantly. This indicated that the compression and deformation of the starch crystalline region caused by the extrusion pressure generated by large ice crystals, resulting in a decrease in the ordering of the crystalline region, a breakage in the double helix hydrogen bond, and a decrease in the relative crystallinity of starch. Compared to the control group, the relative crystallinity of starch was significantly increased with ScAFP added, which may be due to the fact that ScAFP reduced or slowed down the compression damage of ice crystals on the crystalline structure of starch by binding to ice crystals, thus delaying the decrease in the relative crystallinity of starch.

### 3.4 Solubility and swelling power

The solubility and swelling power of starch with or without ScAFP during FTs were investigated, as shown in Table 2. The solubility of starch with or without ScAFP was significantly increased during the FTs compared to natural starch, indicating that the damage degree of ice crystals to starch granules increased and the heat stability of starch granule decreased. The solubility of starch without ScAFP after 10 FTs reached 7.03 %, which may be associated with the disordering of crystalline structure and breakage of intermolecular and intramolecular hydrogen bonds (section 3.3), further causing the dissolved of amylopectin and thus the solubility increased significantly (Liu et al., 2017). After 2 FTs, compared with control, the solubility of starch-ScAFP significantly increased. As is known, ScAFP could reduce the damage of ice crystals to starch granule and inhibit the leakage of free substances inside the starch, thus leading to more soluble substance from starch when heated at 85℃. After 4, 6 and 8 FTs, the addition of ScAFP did not significantly affect the solubility of starch. After 10 FTs, the solubility of starch-ScAFP was significantly lower than that of control, which demonstrated that ScAFP reduced or delayed the damage of ice crystals to the crystalline structure of starch (section 3.3), and accordingly, the hydration ability of starch decreased when heated at 85 °C.

**Table 2:** Solubility and swelling power of starch upon different FTs with and without ScAFP.

Samples	Solubility (%)	Swelling power (%)
NWS	5.34 ± 0.35^a^	9.04 ± 0.17^a^
CS-2c	5.82 ± 0.20^b^	9.59 ± 0.20^bc^
CS-4c	6.15 ± 0.33^bc^	9.58 ± 0.30^bc^
CS-6c	6.33 ± 0.34^cd^	9.78 ± 0.31^cd^
CS-8c	6.11 ± 0.18^bc^	10.03 ± 0.13^d^
CS-10c	7.03 ± 0.27^e^	9.77 ± 0.12^cd^
SS-2c	6.65 ± 0.15^de^	9.87 ± 0.21^cd^
SS-4c	5.99 ± 0.30^bc^	9.62 ± 0.17^bc^
SS-6c	6.24 ± 0.08^bcd^	9.32 ± 0.16^ab^
SS-8c	6.35 ± 0.19^cd^	10.06 ± 0.03^d^
SS-10c	5.88 ± 0.27^bc^	9.71 ± 0.03^cd^

Mean of three measurements ± standard deviation. Different lowercase letters in the same column indicate significant differences (*P* < 0.05) between different treatments. FTs: freeze-thawed cycles; NWS: native wheat starch; CS and SS represent the starch without and with ScAFP added, respectively; c in the first column represents freeze–thaw cycles, and different numbers represent the corresponding number of freeze–thaw cycles.

The swelling power of starch reflects the change of water holding capacity of starch granules without shear behavior, which is closely related to the properties of amylopectin. Compared with native starch, the swelling power of starch with or without ScAFP addition increased significantly during FTs, which was consistent with the results of previous studies (Liu et al., 2022, Tao et al., 2018). This may be due to the mechanical extrusion of ice crystals on the starch granules led to the leaching of amylose, lipids and proteins inside the starch, which promoted the free swelling of amylopectin, and then showed a large swelling power (Liu et al., 2022). After 6 freeze-thawed cycles, it was found that the swelling force of starch with ScAFP was significantly reduced compared with that of control group, which may be attributed to the combination of ScAFP with ice crystals to change the morphology of ice crystals (Cao et al., 2020), thus reducing the damage of ice crystals to starch granules, decreasing the leaching of free material in amorphous areas, and thus inhibiting the swelling of amylopectin.

### 3.5 Pasting properties

The pasting properties and curves of starch with or without ScAFP added during FTs were shown in Table 3 and Fig. S1, respectively. After 2, 4 and 6 FTs, the peak viscosity of starch increased significantly. According to our finding in 3.1, 3.3, 3.4, large ice crystals caused by ice recrystallization compressed and damaged the starch particles, resulting in the appearance of cave and holes on the starch particles, which promoted water infiltration and leaching of free material from the amorphous region of starch, leading to increased swelling of starch and therefore increased peak viscosity. After 8 FTs, the starch amorphous region was compressed, amylopectin rearranged and the proportion of crystalline region increased (section 3.3), which made the granules to expand to greater extent and reach higher viscosity (Srichuwong, Sunarti, Mishima, Isono, & Hisamatsu, 2005). It's worth noting that the peak viscosity of starch decreased after 10 FTs. This was due to, after 10 FTs, starch granules were seriously compressed and destroyed into the crystallization region of starch, leading to the fracture of amylopectin side chain inside the starch and weakening the hydrogen bond association of amylopectin during starch gelatinization, leading to a decrease in peak viscosity (Watcharatewinkul, Puttanlek, Rungsardthong, & Uttapap, 2009). The peak viscosity of starch-ScAFP after freeze-thawed treatment was higher than that of control. This may be due to the fact that ScAFP reduced the compression of ice crystals on the starch granules, resulting in more integrated and stable starch granules that are able to hold more water and have higher peak viscosity during heating.

**Table 3:** Pasting properties and gelatinization parameters of starch upon different FTs with and without ScAFP.

Samples	Pasting properties				Gelatinization parameters			
	Peak viscosity (cP)	Breakdown viscosity (cP)	Setback viscosity (cP)		T_o_ (℃)	T_p_ (℃)	T_c_ (℃)	ΔH (J/g)
NWS	2211 ± 60^a^	343 ± 19^a^	462 ± 10^a^		52.68 ± 0.18^a^	61.20 ± 0.02^bc^	67.60 ± 0.11^a^	6.71 ± 0.21^a^
CS-2c	2420 ± 25^b^	395 ± 10^e^	600 ± 13^e^		53.72 ± 0.15^ef^	61.11 ± 0.09^abc^	68.50 ± 0.32^b^	7.70 ± 0.14^bc^
CS-4c	2406 ± 68^b^	376 ± 6^cde^	585 ± 14^de^		53.42 ± 0.30^cde^	61.23 ± 0.15^bc^	67.59 ± 0.22^a^	7.60 ± 0.30^bc^
CS-6c	2395 ± 14^b^	367 ± 2^cd^	565 ± 20^d^		53.97 ± 0.26^f^	61.48 ± 0.07^d^	67.59 ± 0.50^a^	7.40 ± 0.05^b^
CS-8c	2484 ± 25^c^	386 ± 2^de^	596 ± 10^de^		54.4 ± 0.33 ^g^	61.81 ± 0.05^e^	68.55 ± 0.45^b^	7.60 ± 0.07^bc^
CS-10c	2367 ± 36^b^	356 ± 1^abc^	514 ± 27^bc^		52.86 ± 0.21^ab^	61.03 ± 0.03^ab^	67.64 ± 0.30^a^	7.40 ± 0.09^b^
SS-2c	2523 ± 15^cd^	366 ± 10^cd^	485 ± 20^ab^		53.17 ± 0.22^bcd^	61.28 ± 0.12^cd^	69.81 ± 0.23^d^	7.80 ± 0.20^c^
SS-4c	2543 ± 22^cd^	344 ± 15^ab^	516 ± 25^bc^		53.32 ± 0.30^cde^	60.94 ± 0.21^a^	68.6 ± 0.13^b^	7.59 ± 0.19^bc^
SS-6c	2559 ± 4^d^	365 ± 3^bcd^	529 ± 18^c^		53.07 ± 0.10^abc^	61.10 ± 0.19^abc^	68.55 ± 0.33^b^	7.36 ± 0.30^b^
SS-8c	2702 ± 40^e^	384 ± 16^de^	511 ± 10^bc^		53.57 ± 0.12^def^	61.50 ± 0.14^d^	68.75 ± 0.30^bc^	7.57 ± 0.20^bc^
SS-10c	2505 ± 33^cd^	359 ± 19^abc^	475 ± 22^a^		53.27 ± 0.25^bcd^	61.13 ± 0.15^abc^	69.15 ± 0.00^c^	7.77 ± 0.13^c^

Mean of three measurements ± standard deviation. Different lowercase letters in the same column indicate significant differences (*P* < 0.05) between different treatments. The onset temperature (T_o_), the endothermic peak temperature (T_p_), the conclusion temperature (T_c_) and the enthalpy of gelatinization (ΔH, J/g). FTs: freeze-thawed cycles; NWS: native wheat starch; CS and SS represent the starch without and with ScAFP added, respectively; c in the first column represents freeze–thaw cycles, and different numbers represent the corresponding number of freeze–thaw cycles.

The breakdown viscosity, which represents the difference between peak viscosity and trough viscosity, reflects the anti-shear abilities of granules during heating. The higher breakdown viscosity suggests that starch is difficult to withstand shear force and is easily destroyed during heating (Su et al., 2020). After the freeze-thawed treatment, the breakdown viscosity of starch was significantly higher than that of native starch, suggesting that the freeze-thawed starch was more easily destroyed during heating. Compared with control, the breakdown viscosity of starch-ScAFP after 2 and 4 FTs was significantly lower, while after 6, 8 and 10 FTs, the addition of ScAFP had no significant influence on the breakdown viscosity, indicating that ScAFP could delay the reduction of shear resistance of starch induced by FTs.

Setback viscosity reflects the short-term retrogradation of starch (Liu et al., 2017). After FTs, the setback viscosity of starch increased significantly, indicating that the freeze-thawed starch had a greater tendency for retrogradation. Su et al. (2020) has reported the setback viscosity of starch after 10 weeks frozen storage (-80 °C) was higher than native starch, and Tao et al. (2015) found the setback viscosity of starch after 7 and 10 FTs was higher than native starch. After frozen storage or FTs, the amylose was leaked and realigned to form a well-organized double helix structure frame, resulting in increased setback viscosity of starch (Su et al., 2020). Compared with 2, 4, 6, and 8 FTs, the setback viscosity of starch significantly reduced after 10 FTs, which was probably associated with the disruption on the crystalline structure of starch by ice crystals (section 3.3). However, compared with control, the setback viscosity of freeze-thawed starch-ScAFP decreased significantly, indicating that ScAFP could inhibit the increase in starch retrogradation caused by freeze-thawed treatment.

### 3.6 Thermal properties

The gelatinization parameters of starch with or without ScAFP added during multiple FTs were shown in Table 3. The outer chains of amylopectin in starch gather together in the form of double helix clusters to form crystallites, which combine to form ordered regions or “crystal lamellae” (Tao, Yan, Zhao, Tian, Jin, & Xu, 2015). Starch gelatinization refers to the unwinding and melting of amylopectin outer chain in the crystalline region. Therefore, gelatinization temperature (T_o_, T_p_, T_c_) and gelatinization enthalpy of starch represent the order degree of double helix and its crystallinity degree (Zhang et al., 2021).

After 2, 4, 6, and 8 FTs, T_o_ of starch increased significantly, showing that starch had a more orderly crystalline structure. This may be because large ice crystals formed destroyed the granular structure of starch, leading to the leaching of amylose, amylopectin rearrangement and formation of a well-organized double helix structure frame (Tao et al., 2015). Compared to the starch after 8 FTs, the gelatinization temperature of starch after 10 FTs decreased significantly, which was due to the weakening of the ordered structure in the starch as the large ice crystals formed after 10 FTs disrupted the crystalline structure of the starch (section 3.3). After the addition of ScAFP, compared with the control group, the T_o_ of freeze-thawed starch decreased significantly after the 2nd, 6th and 8th FTs, while the T_c_ of freeze-thawed starch increased significantly. This may be because ScAFP inhibits the recrystallization of ice crystals and reduces the damage of ice crystals to starch particles, thus reducing the leaching of amorphous materials and possibly inhibiting the amylopectin rearrangement in the crystalline region. Therefore, the order of double helix structure is reduced, and the uniformity of the crystal structure of starch is poor.

The gelatinization enthalpy of starch is a reflection of the energy needed to destroy the ordered structure of starch during gelatinization (Su et al., 2020). After freeze-thawed treatment, the gelatinization enthalpy of starch increased, which was attributed to the rearrangement of the internal double helix structure of starch resulted in a more ordered and stable crystalline structure, thus increasing the energy needed to melt the ordered structure of starch during heating, which was in agreement with the results of previous studies (Tao et al., 2018, Tao et al., 2015). Compared with control, the gelatinization enthalpy of freeze-thawed starch-ScAFP significantly increased after 10 FTs, which was due to ScAFP reduced the damage of ice crystals to the crystalline structure of starch, resulting in significant increase in starch gelatinization energy. After the addition of ScAFP, the gelatinization enthalpy of freeze-thawed starch did not change significantly after the 2nd, 4th, 6th and 8th FTs compared with the control group. This may be because ice crystals have little effect on the ordered structure of starch at this stage, so the addition of ScAFP showed little effect on the gelatinization enthalpy of starch. After the 10th FTs, the gelatinization enthalpy of starch increased significantly. This may be related to the remarkable property of ScAFP being able to bind to the surface of ice crystals, which greatly limited the growth and inhibited recrystallization of ice crystals, thus reducing or delaying the disruption of the crystalline structure of starch by ice crystals (Tirado-Kulieva et al., 2022), which was in line with the test results of XRD.

### 3.7 Gel texture properties

The gel texture properties of starch with or without ScAFP during FTs were concluded in Table 4. Gel hardness indicates the force required for the sample to produce deformation (Guo et al., 2020). Compared with native starch, the gel hardness of starch after 2 FTs significantly reduced. This may be attributed to the mechanical damage of ice crystals to starch granules during 2 FTs, which led to the leaching of amylose and short-chain amylopectin in the amorphous region of starch. This caused a decrease in the substances which participate in the formation of three-dimensional network after starch gelatinization and a reduction in the crosslinking degree between starch molecules, which in turn led to the lower hardness of starch gels. The gel hardness of starch after 6 FTs was higher than that of starch after 2 FTs. Due to the damage of starch granules by ice crystals, more channels in which water permeate were formed inside the starch granule and internal ice crystals grew up. The expansion of internal ice crystals promoted the amorphization of ordered molecular structure, leading to the leaching of more amylopectin during pasting, and subsequently the cross-linking degree of starch gel network was enhanced. After 10 FTs, the gel hardness of starch significantly decreased. Ice crystals seriously destroyed the amorphous region and crystalline structure of starch, and led to the fracture of more starch chain and leaching of amylopectin during 10 FTs, eventually bringing about a failure to form a compact gel network after starch gelatinization. The gel texture properties of starch were affected by starch interaction and swelling behavior during heating process, which were closely related to the pasting characteristics and swelling power (Waterschoot, Gomand, Willebrords, Fierens, & Delcour, 2014).

**Table 4:** Gel texture properties of starch upon different FTs with and without ScAFP.

Samples	Hardness (g)	Adhesiveness (g s)	Springiness	Chewiness
NWS	368.04 ± 8.13^ef^	354.58 ± 0.02^h^	0.902 ± 0.002^a^	134.23 ± 3.53^ab^
CS-2c	347.39 ± 13.01^cd^	321.05 ± 10.12^f^	0.958 ± 0.002^e^	141.04 ± 3.14^abcd^
CS-4c	354.62 ± 6.68^de^	227.89 ± 9.78^d^	0.969 ± 0.006^f^	146.89 ± 2.30^de^
CS-6c	366.89 ± 12.61^ef^	206.98 ± 8.40^c^	0.949 ± 0.001^d^	154.28 ± 6.04^e^
CS-8c	330.25 ± 0.63^b^	192.07 ± 9.51^d^	0.966 ± 0.001^ef^	142.27 ± 3.36^bcd^
CS-10c	302.34 ± 5.43^a^	156.28 ± 2.36^a^	0.968 ± 0.006^f^	132.83 ± 4.99^a^
SS-2c	397.97 ± 10.92^g^	158.28 ± 10.12^a^	0.967 ± 0.003^f^	176.92 ± 10.36^f^
SS-4c	380.08 ± 5.30^f^	308.85 ± 11.21^f^	0.941 ± 0.005^d^	147.75 ± 0.22^de^
SS-6c	382.42 ± 3.80^f^	368.27 ± 1.03^h^	0.911 ± 0.001^b^	145.51 ± 3.37^cde^
SS-8c	371.72 ± 11.18^f^	334.92 ± 10.14^g^	0.921 ± 0.003^c^	142.70 ± 3.04^bcd^
SS-10c	334.11 ± 11.55^bc^	277.03 ± 5.31^e^	0.947 ± 0.011^d^	136.80 ± 5.75^abc^

Mean of three measurements ± standard deviation. Different lowercase letters in the same column indicate significant differences (*P* < 0.05) between different treatments. FTs: freeze-thawed cycles; NWS: native wheat starch; CS and SS represent the starch without and with ScAFP added, respectively; c in the first column represents freeze–thaw cycles, and different numbers represent the corresponding number of freeze–thaw cycles.

The gel hardness of starch-ScAFP was significantly higher than that of control. Gong et al. (2019) and Cheng et al. (2002) had shown that hydrogen bonds formed between the ice surface and antifreeze proteins could distort the ice surface, modified ice crystal morphology and inhibited ice recrystallization, which in turn reduced the mechanical damage caused by ice crystals to frozen food and improved product quality. Therefore, ScAFP could effectively reduce starch damage caused by ice crystals and improve the gel hardness of starch.

Adhesiveness is mainly associated with the combined impacts of adhesive and cohesive forces (Zhang et al., 2023). After FTs, the gel adhesiveness of starch-control significantly decreased, and gradually decreased with FTs increasing. Compared with starch-control, the adhesiveness of starch-ScAFP significantly increased after 4, 6, 8, and 10 FTs. The chewiness is correlated with the gel network structure, and higher chewiness value indicates more energy is required to chew the gel (Guo et al., 2020). The chewiness of starch gel firstly increased and then decreased as FTs increased, with the maximum value reached after 6 FTs. The chewiness of starch-ScAFP increased significantly after 2 FTs and did not significantly change after 4, 6, 8, and 10 FTs. Considering these three indicators (hardness, adhesiveness, and chewiness) together, FTs destructed the texture properties of starch gel and the addition of ScAFP significantly enhanced its texture properties.

## 4 Conclusions

In this study, the effects of FTs and ScAFP on the microstructure observation, particle size distribution, relative crystallinity, solubility, swelling power, pasting properties, thermal properties and gel texture properties of wheat starch were studied. Mechanical damage to starch granules by ice crystals gradually deepened with FTs increasing. Firstly, starch granules were squeezed by ice crystals, resulting in surface damage of starch granules; starch amorphous region was compressed and more internal material leached out as FTs increased; afterwards, the ordered structure of starch crystallization region was extruded and deformed lea This may be attributed to the inhibition of ice to its tendency to disorder. The weakening of the starch granule structure further leaded to changes in its physicochemical properties, including reduced thermal stability, increased retrogradation tendency, and weakened gel network structure. The addition of ScAFP retarded the damage of ice crystals on starch granule structure and crystal structure during FTs, significantly reduced the retrogradation tendency and improved the gel texture properties of freeze-thawed starch. Therefore, ScAFP is expected to be an effective cryoprotectant to reduce the quality deterioration of starch-based foods caused by ice crystals during FTs. Next, the multi-scale structure of starch (chain, amorphous and crystalline lamellae, blocklets, etc.) will be further investigated to more systematically elucidate the improvement mechanism of ScAFP on freeze–thaw starch.

## CRediT authorship contribution statement

**Mei Liu:** Conceptualization, Writing – original draft, Methodology, Supervision. **Jie Li:** Conceptualization, Formal analysis, Writing – review & editing. **Hao Ma:** Methodology, Formal analysis, Investigation. **Guolan Qin:** Formal analysis. **Mengge Niu:** Investigation, Visualization. **Xiaoyin Zhang:** Investigation, Visualization. **Jin Zhang:** Investigation. **Yangkun Wei:** Investigation. **Jiajing Han:** Visualization. **Ying Liang:** Writing – review & editing, Investigation. **Shenying Zhang:** Visualization. **Lulu Yin:** Methodology. **Haojia Zhu:** Methodology. **Ying Huang:** Methodology. **Limin Li:** Methodology, Resources. **Xueling Zheng:** Supervision, Project administration. **Chong Liu:** Writing – review & editing.

## Declaration of Competing Interest

The authors declare that they have no known competing financial interests or personal relationships that could have appeared to influence the work reported in this paper.

## Data Availability

The authors do not have permission to share data.
