# Supplementary figures and images for: Structural and physicochemical characteristics of wheat starch as influenced by freeze-thawed cycles and antifreeze protein from Sabina chinensis (Linn.) Ant. cv. Kaizuca leaves
Source: Food Chem X. 2023 Oct 5;20:100927. doi: 10.1016/j.fochx.2023.100927 (PMC10740099; doi:10.1016/j.fochx.2023.100927)

# Figure captions

Fig. S1. Pasting curves of starch with and without ScAFP during different FTs.


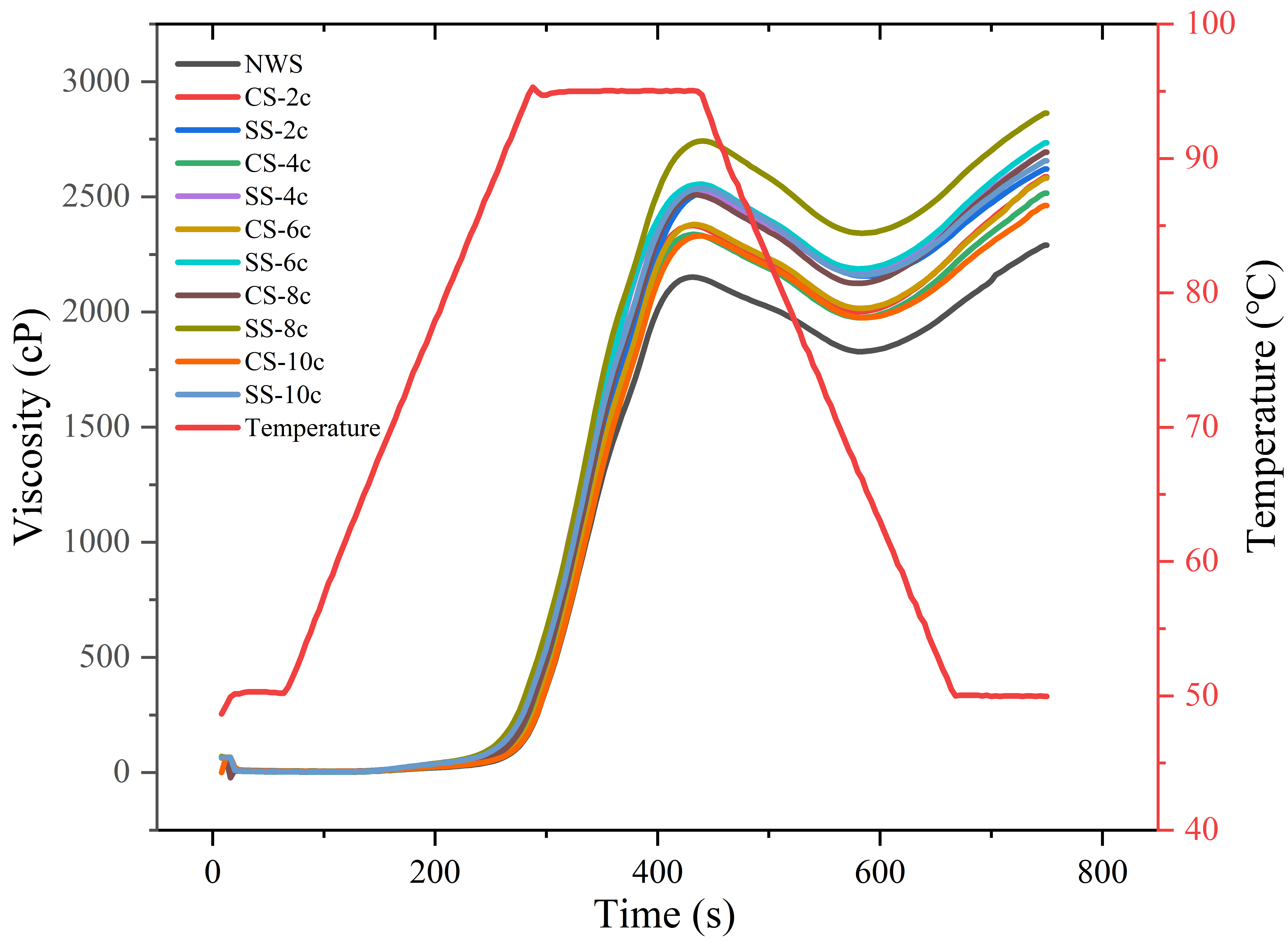


Fig. 1

Supplement: Supplementary data 1 [file mmc1.docx]
